# Impact of tumor burden on prognostic prediction for patients with terminal stage hepatocellular carcinoma: A nomogram study

**DOI:** 10.1371/journal.pone.0188031

**Published:** 2017-11-10

**Authors:** Chia-Yang Hsu, Po-Hong Liu, Shu-Yein Ho, Yi-Hsiang Huang, Yun-Hsuan Lee, Yi-You Chiou, Ting-Hui Hsieh, Tom Fang, Ya-Ju Tsai, Ming-Chih Hou, Teh-Ia Huo

**Affiliations:** 1 Department of Medicine, Taipei Veterans General Hospital, Taipei, Taiwan; 2 Faculty of Medicine, National Yang-Ming University School of Medicine, Taipei, Taiwan; 3 Department of Internal Medicine, University of Nevada School of Medicine, Reno, NV, United States of America; 4 Harvard T.H. Chan School of Public Health, Boston, MA, United States of America; 5 Institute of Clinical Medicine, National Yang-Ming University School of Medicine, Taipei, Taiwan; 6 Department of Radiology, Taipei Veterans General Hospital, Taipei, Taiwan; 7 Gastroenterology Consultants, Reno, NV, United States of America; 8 Renown Regional Medical Center, Reno, NV, United States of America; 9 Institute of Pharmacology, National Yang-Ming University School of Medicine, Taipei, Taiwan; Yonsei University College of Medicine, REPUBLIC OF KOREA

## Abstract

**Background:**

The recently proposed nomogram of Barcelona Clinic Liver Cancer (BCLC) lacks predictive accuracy for patients with stage D hepatocellular carcinoma (HCC). Tumor burden is crucial in prognostic prediction but is not included in the criteria of stage D HCC. This study aims to develop a nomogram with tumor burden as the core element for BCLC stage D patients.

**Methods:**

A total of 386 patients were randomly grouped into derivation and validation sets (1:1 ratio). The multivariate Cox proportional hazards model was used to select factors with significant prognostic effect and generate the nomogram. Concordance indices and calibration plots were used to evaluate the performance of nomogram.

**Results:**

Overall survival of study patients was significantly associated with tumor burden as well as hepatitis B, serum α-fetoprotein level, cirrhosis and performance status in multivariate Cox regression (all p<0.05). Beta-coefficients of these variables in derivation set were used to generate the nomogram. Each patient was assigned with a total nomogram point that predicted individualized 6-month and 1-year survival. The derivation and validation sets had a c-index of 0.759 (95% confidence interval [CI]: 0.552–0.923) and 0.741 (95% CI: 0.529–0.913), respectively. The calibration plots were close to the 45-degree line for 6-month and 1-year survival prediction for all quarters of patients in both derivation and validation sets.

**Conclusion:**

Tumor burden is significantly associated with the outcome for patients with stage D HCC. The tumor burden-incorporated nomogram may serve as a feasible and easy-to-use tool in predicting survival on an individual level.

## Introduction

Hepatocellular carcinoma (HCC) is the most common primary liver cancer in the world. Major academic societies of liver disease recommend the Barcelona Clinic Liver Cancer (BCLC) staging system to be the prognostic model and allocating tool for treatment selection.[1, 2] Three major parameters including tumor burden, severity of cirrhosis and performance status (PS) have been used to predict the prognosis of HCC. Patients with Child-Turcotte-Pugh (CTP) class C or PS 3–4 are classified as terminal stage, or stage D, HCC because of very limited survival time after diagnosis with or without anti-cancer treatments. Tumor burden, including size and number of tumor nodule(s), vascular invasion and extra-hepatic involvement, may profoundly influence the outcome of HCC patients; however, it is not considered a criterion for BCLC stage D.[3, 4] So far, there is no comprehensive investigation regarding the prognostic effect of tumor burden for stage D HCC patients.

Recently, the nomogram of BCLC system, which provides individualized prediction of patient survival, has been proposed and externally validated.[5–7] The nomogram is a straightforward tool and does not require additional laboratory or imaging studies to accurately predict patient outcome except for those with BCLC stage D HCC.[7] The estimation of survival for stage D patients may need to be specifically designed because of their extremely poor prognosis resulting from advanced cirrhosis and/or debilitated general condition as well as various tumor burden (from a single small nodule to distant metastases). In addition, establishing an accurate prognostic model for late cancer stage has always been important for patients considering hospice care.[8, 9] Furthermore, the emergence and applications of immunotherapy show possible survival benefits for HCC, which also demands a feasible survival-predicting tool before large-scale clinical trials can be planned.[10, 11] This study aimed to investigate if tumor burden is related to the overall outcome in patients with terminal stage HCC, and to customize a nomogram for better prognostic stratification.

## Patients and methods

### Patients

During a 14-year period between 2002 and 2016, 386 newly diagnosed BCLC stage D patients in our hospital were prospectively collected and retrospectively analyzed. Etiology of underlying liver disease, number and size of tumor(s), serum biochemistry, PS, and liver cirrhosis were comprehensively recorded at the time of diagnosis. The survival status of all patients was checked every 3–4 months after enrollment and was confirmed by using the database of National Cancer Registry, Taiwan. Part of the study patients had been reported as described in our previous study.[7] This study complies with the standards of the Declaration of Helsinki and current ethical guidelines, and has been approved by the institutional review board (IRB; protocol number 2016-04-005AC) of Taipei Veterans General Hospital, Taiwan. The waiver of consent was obtained as justified by the IRB, and patient records/information was anonymized and de-identified prior to analysis.

### Diagnosis and definitions

Findings of typical radiological features in at least two imaging modalities including contrast-enhanced dynamic computed tomography (CT), magnetic resonance imaging (MRI), ultrasound and hepatic arterial angiography, or by a single positive imaging study associated with serum α-fetoprotein (AFP) level ≥ 400 ng/mL or histological confirmation were used to diagnose HCC.[12] Patients who were seropositive for anti-hepatitis C virus (HCV) antibody were classified as HCV-related HCC. Hepatitis B virus (HBV)-related HCC was defined as seropositive for hepatitis B surface antigen. Daily consumption of at least 40 g of alcohol for 5 years or more was considered alcoholic liver disease.[13] Vascular invasion was diagnosed by the presence of thrombus adjacent to the tumor in portal system by at least two imaging modalities. Total tumor volume was calculated based on tumor diameter of every HCC nodule as previously described.[14] The Eastern Cooperative Oncology Group (ECOG) criteria were used to evaluate the overall physical status of study patients at the time of diagnosis.[15] Patients who are fully active were recorded as PS 0. Patients with some restriction of activity but still able to carry out work were documented as PS 1. Patients are ambulatory but unable to do any work activity were considered PS 2. Patients are capable of limited self-care and confined to bed or chair more than 50% of waking hours were classified as PS 3. Patients who are completely disabled and totally confined to bed or chair were recorded as PS 4. Patients with tumor burden within the Milan criteria (one nodule < 5 cm, or up to 3 nodules < 3 cm without vascular invasion or extra-hepatic involvement) were classified as tumor burden grade 1.[16] Patients were recorded as tumor burden grade 3 if lymph node involvement, vascular invasion, or distant metastasis were confirmed at the time of diagnosis. All remaining patients were coded as tumor burden grade 2. Chest CT scan was performed to detect metastatic lesion(s) and lymph node involvement. Bone metastasis from HCC was surveyed by bone scan and confirmed by MRI if indicated. All clinical data were recorded at the time of diagnosis.

### Statistics

Categorical data were compared with the chi-squared or Fisher exact tests. Continuous characteristics were compared with the Mann-Whitney ranked sum test. The comparison of survival distributions was performed by using the Kaplan-Meier method with a log-rank test. All HCC-related variables were tested by the univariate survival analysis; variables with significant effect on prognosis were introduced into the multivariate Cox proportional regression model to generate beta coefficients (BETA). The ratios of calculated BETAs were used to determine the proportional prognostic effect in the nomogram. The efficiency of the nomogram model was examined by the concordance index,[17, 18] which estimates the probability that for two randomly selected patients, when one patient has an event after the other, this patient has fewer total points by the nomogram. Calibration was conducted by comparing the mean of nomogram-calculated survival with the survival distribution observed by the Kaplan-Meier method. A p value less than 0.05 was considered statistically significant. All statistical analyses were conducted with the SAS 9.4 (SAS Institute Inc., Cary, NC, USA).

## Results

### Patient characteristics

The baseline demographics of study patients are shown in Table 1. The mean age of enrolled patients was 66 years, and 22% of them were female. Hepatitis B (49%) was the predominant etiology of chronic liver disease, followed by hepatitis C and alcoholism. Forty-nine percent of patients had multiple tumors, and 70% of patients had a primary tumor diameter larger than 5 cm. There were 17%, 43% and 41% of patients who were classified as CTP class A, B, C respectively, and 0.25%, 9%, 7%, 55%, and 29% of patients had PS 0, 1, 2, 3, 4, respectively. Vascular invasion was found in 56% of patients, and 28% of patients had diabetes mellitus. A total of 103 (27%) patients were confirmed to have distant metastasis or lymph node involvement at diagnosis.

**Table 1:** Baseline demographics.

Number of patients	386
Age (years, mean±standard deviation [SD])	66 ± 15
Male/female (%)	78/22
Etiology of cirrhosis (%)	
Hepatitis B	191 (49)
Hepatitis C	112 (29)
Alcoholism	88 (23)
Serum biochemistry (mean±SD)	
Albumin (g/dL)	2.9 ± 0.6
Bilirubin (mg/dL)	4.4 ± 6
Creatinine (mg/dL)	1.4 ± 1.2
Estimated glomerular filtration rate (ml/min/1.73m^2^)	69 ± 41
International normalized ratio of prothrombin time	1.3 ± 0.3
Child-Turcotte-Pugh class A/B/C (%)	17/43/41
Number and size of tumor (%)	
Single/multiple	51/49
≤ 5 cm/ > 5 cm	30/70
Total tumor volume (cm^3^, mean±SD [median])	685 ± 1,055 (324)
Vascular invasion (%)	215 (56)
Metastasis/lymph node	103 (27)
α-fetoprotein (ng/mL, mean±SD [median])	359,623 ± 235,826 (441)
Tumor burden 1/2/3 (%)	15/20/65
Ascites (%)	280 (73)
Performance status 0/1/2/3/4 (%)	0.25/9/7/55/29
Diabetes mellitus (%)	107 (28)
Treatment modality (%)	
Resection	13 (3)
Transplantation	6 (2)
Ablation	29 (8)
Transarterial chemoembolization	53 (14)
Targeted therapy (sorafenib)	13 (3)
Best supportive care	272 (70)

For the anti-cancer treatments, 3% of patients received surgical resection, and 2%, 8%, 14%, 3% and 70% of patients underwent transplantation, local ablation, transarterial chemoembolization (TACE), targeted therapy (sorafenib) and best supportive care, respectively.

### Survival distribution of patients stratified by tumor burden

**Fig 1:**
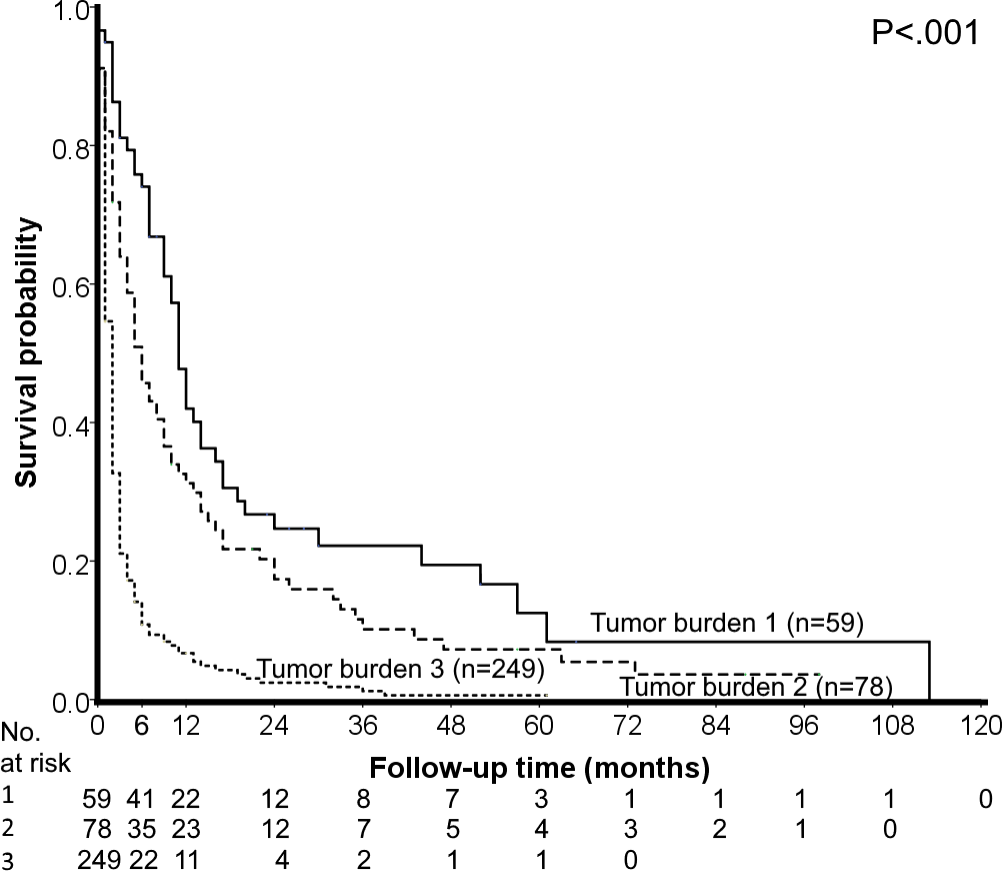
Survival distribution according to tumor burden for all patients. The survival of patients with smaller tumor burden is significantly better than that of patients with larger/more tumor nodule(s).

After an average follow-up period of 7.4 (median, 2) months, 349 (90%) patients died. As shown in Fig 1, patients with larger tumor burden had significantly worse overall survival (p< 0.001).

### Univariate and multivariate survival analyses

Variables that were possibly linked with survival were investigated by using the Kaplan-Meier method (Table 2). Hepatitis B, alcoholism, larger tumor burden, advanced cirrhosis, poor PS and high serum AFP level were significantly associated with decreased survival of all study patients in univariate analysis (all p< 0.05). The multivariate model confirmed the independent prognostic effect of these variables except for alcoholism.

**Table 2:** Univariate and multivariate survival analyses.

		Univariate analysis of all patients			Multivariate analysis of all patients		Multivariate analysis of derivation set	
	N	6-month survival (%)	1-year survival (%)	P	BETA/hazard ratio	p	BETA/hazard ratio	p
Sex (Male/Female)	301/85	27/32	17/18	0.894				
Age (<66/≥66 years)	191/195	24/32	14/21	0.1				
HBV (Neg/Pos)	195/191	34/23	22/11	0.01	0.252/1.286	0.029	0.343/1.423	0.032
HCV (Neg/Pos)	274/112	28/28	17/19	0.953				
Alcoholism (Neg/Pos)	298/88	30/22	19/11	0.036				
Tumor burden				< .001				
1	59	74	42		0/1		0/1	
2	78	46	31		0.406/1.501	0.036	0.601/1.823	0.036
3	249	11	7		1.095/2.988	< .001	1.064/2.899	< .001
Child-Turcotte-Pugh				< .001				
A	64	47	34		0/1		0/1	
B	165	29	13		0.429/1.536	0.007	0.442/1.556	0.07
C	157	30	15		0.593/1.81	0.001	0.823/2.277	0.003
Performance status				0.014				
0–2	62	49	26		0/1		0/1	
3–4	324	25	16		0.374/1.453	0.044	0.613/1.846	0.023
α-fetoprotein (<400/≥400 ng/mL)	187/199	40/17	28/8	< .001	0.281/1.324	0.016	0.358/1.431	0.032
Diabetes mellitus (Neg/Pos)	279/107	27/31	17/19	0.293				
eGFR (<60/≥60 ml/min/1.73m^2^)	176/210	25/31	16/18	0.087				

BETA, beta coefficient; HBV, hepatitis B virus; HCV, hepatitis C virus; eGFR, estimated glomerular filtration rate

### Characteristics of patients in derivation and validation sets

Patients were randomly split into derivation and validation sets based on 1:1 ratio. Comparison of these two patient groups showed no significant baseline differences (all p> 0.05; Table 3). The derivation group was used to evaluate the prognostic effect of variables which were significantly associated with survival in the univariate analysis to determine the BETAs. Hepatitis B (BETA = 0.343, p = 0.032), tumor burden 2 and 3 compared to tumor burden 1 (BETA = 0.601 and 1.064, p = 0.036 and < 0.001, respectively), CTP class B and C compared to class A (BETA = 0.442 and 0.823, p = 0.07 and 0.003, respectively), PS 3–4 compared to PS 0–2 (BETA = 0.613, p = 0.023), and serum AFP ≥ 400 ng/mL (BETA = 0.358, p = 0.032) were significantly associated with a decreased overall survival (Table 2), which were used to generated the nomogram.

**Table 3:** Comparison of demographics of the derivation and validation sets.

	Derivation set(n = 193)	Validation set (n = 193)	p value
Age (years; mean ± SD)	66 ± 15	66 ± 14	0.959
Age ≥ 66 years	98 (51)	87 (50)	0.262
Male (n, %)	153 (79)	148 (77)	0.539
Liver disease (n, %)			
Hepatitis B	90 (47)	101 (52)	0.263
Hepatitis C	63 (33)	49 (25)	0.116
Alcoholism	42 (22)	46 (24)	0.628
Tumor size > 5 cm (n, %)	141 (73)	131 (68)	0.265
Multiple tumors (n, %)	90 (47)	99 (51)	0.360
Metastasis/lymph node (n, %)	51 (26)	52 (27)	0.908
Total tumor volume (cm3, mean ± SD [median])	772 ± 999 (381)	657 ± 1,110 (279)	0.517
Vascular invasion (n, %)	105 (54)	110 (57)	0.608
α-fetoprotein ≥ 400 ng/mL (n, %)	97 (50)	102 (53)	0.611
CTP class (n, %)			0.214
A	27 (14)	37 (19)	
B	90 (47)	75 (39)	
C	76 (39)	81 (42)	
Ascites (n, %)	139 (72)	141 (73)	0.820
Biochemistry (mean ± SD)			
Albumin (g/dL)	3 ± 0.6	2.9 ± 0.6	0.552
Bilirubin (mg/dL)	4 ± 5.2	4.6 ± 7	0.639
INR of PT	1.3 ± 0.3	1.3 ± 0.3	0.817
eGFR ≥ 60 (mL/min/1.73 m2) (n, %)	107 (55)	103 (53)	0.683
Diabetes mellitus (n, %)	50 (26)	57 (30)	0.426
Performance status 0-2/3-4 (%)	15/85	18/82	0.407
Tumor burden (n, %)			0.170
1	26 (13)	33 (17)	
2	46 (24)	32 (17)	
3	121 (63)	128 (66)	
Treatment (n, %)			0.785
Surgical resection	6 (3)	7 (4)	
Ablation	14 (7)	15 (8)	
Transplantation	2 (1)	4 (2)	
TACE	31 (16)	22 (11)	
Targeted therapy	6 (3)	7 (4)	
Supportive care	134 (69)	138 (72)	

CTP, Child-Turcotte-Pugh; eGFR, estimated glomerular filtration rate; INR, international normalized ratio; PT, prothrombin time; SD, standard deviation; TACE, transarterial chemoembolization

### Construction of the nomogram model

**Fig 2:**
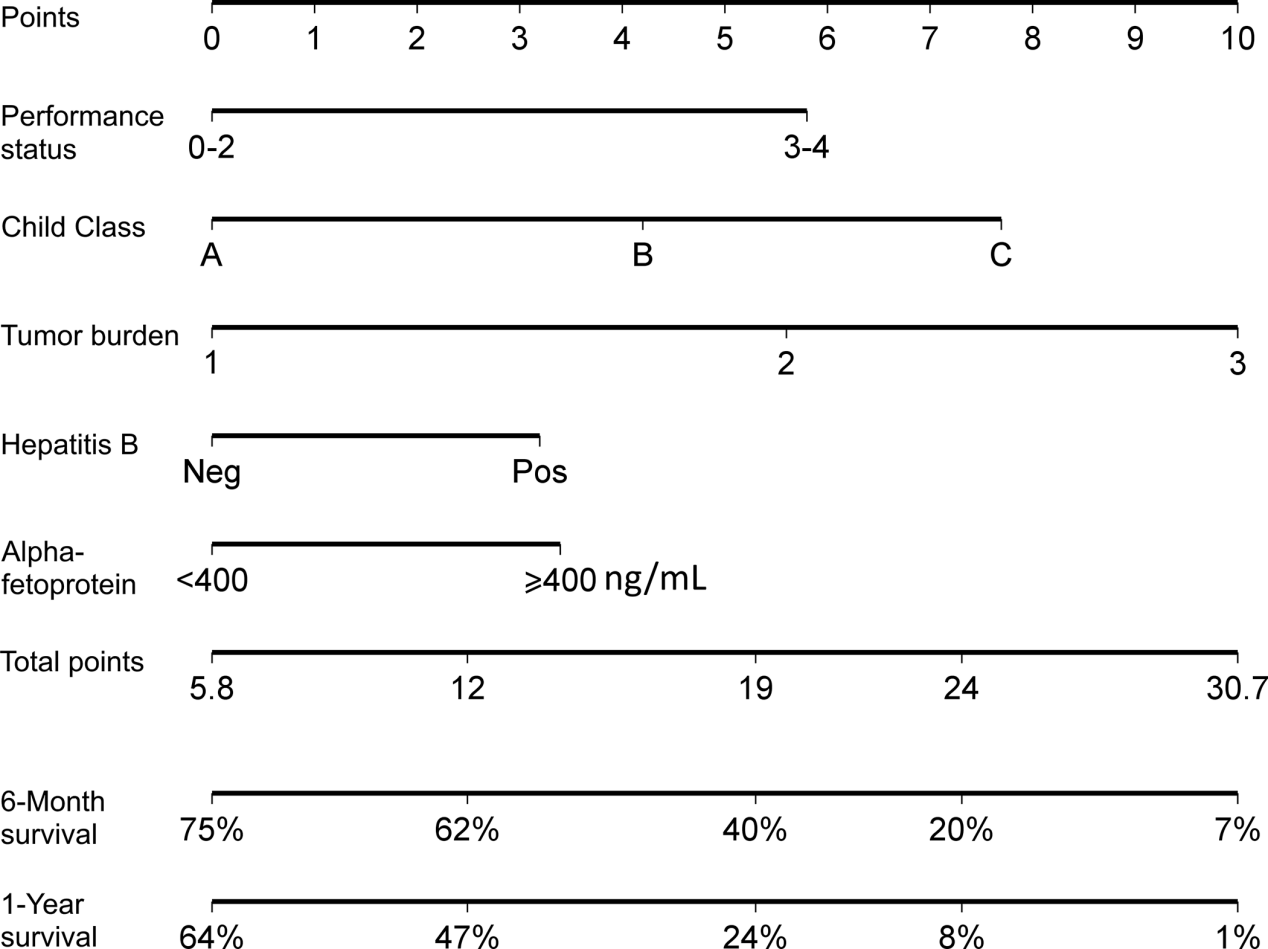
Nomogram predicting 6- and 12-month survival of HCC patients. The nomogram is used by adding up the points identified on the scale for the 5 parameters. The total points project downward to obtain the estimate 6- and 12-month survival.

**Fig 3:**
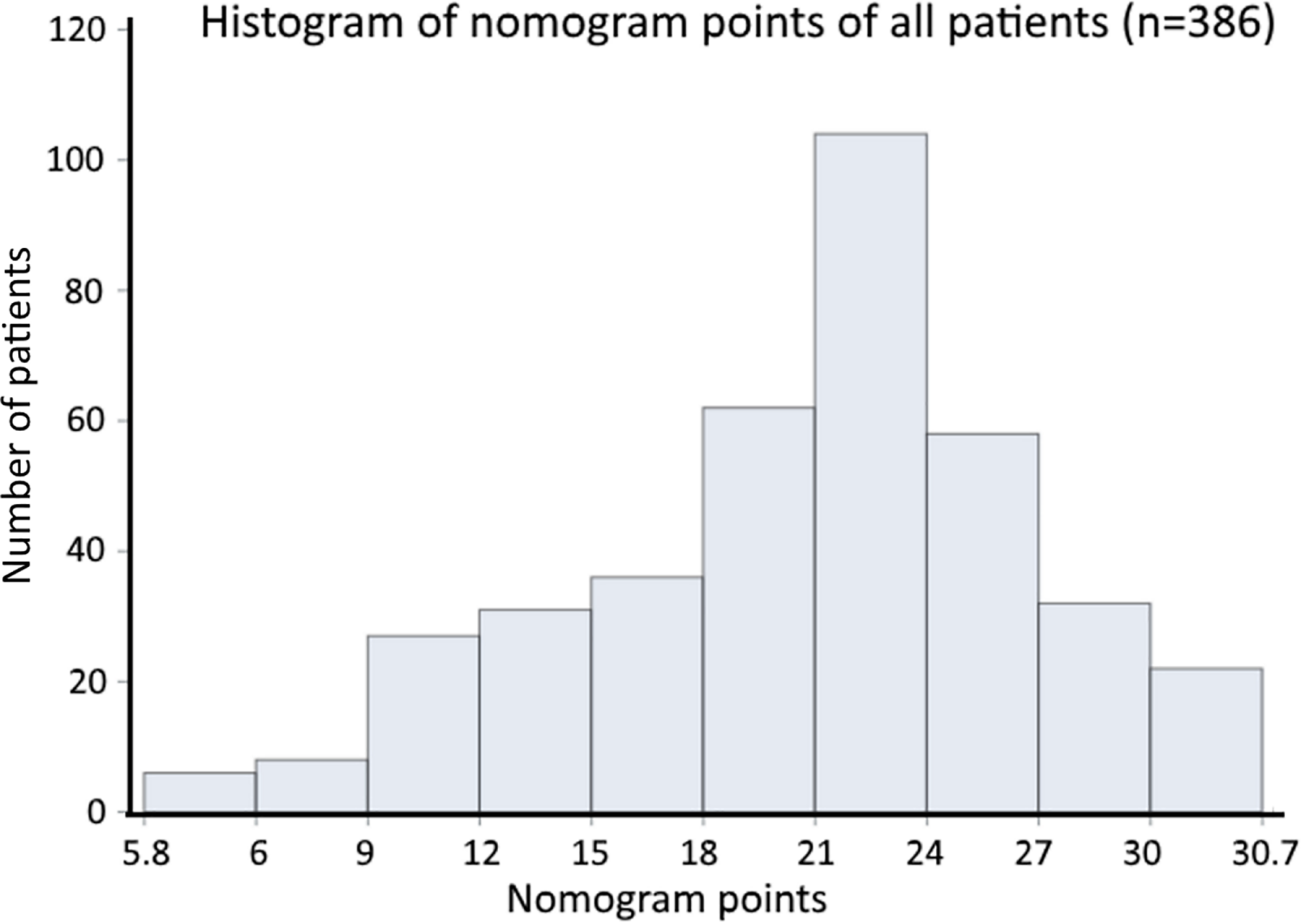
The histogram of nomogram points of all enrolled patients.

Tumor burden 3 had the highest BETA value in the model and was set as 10 points (Table 2). Sequentially, by using the ratios of BETAs between other prognostic factors and tumor burden 3, 7.7 (calculated as 0.823 divided by 1.064 and timed 10), 4.2, 5.6, 3.2, 5.8, 3.4 points were assigned to patients who were CTP class C, CTP class B, tumor burden 2, hepatitis B, PS 3–4 and AFP ≥ 400 ng/mL, respectively. Each patient had one individualized score from 5.8 to 30.7 by adding up the points from these five prognostic predictors. As shown in Fig 2, the projections from total points on the scales below indicate the estimated survival probability at 6 and 12 months. The histogram shows the majority of patients had a nomogram point between 18 to 27 (Fig 3).

### Discrimination and calibration of nomogram in the derivation set

**Fig 4:**
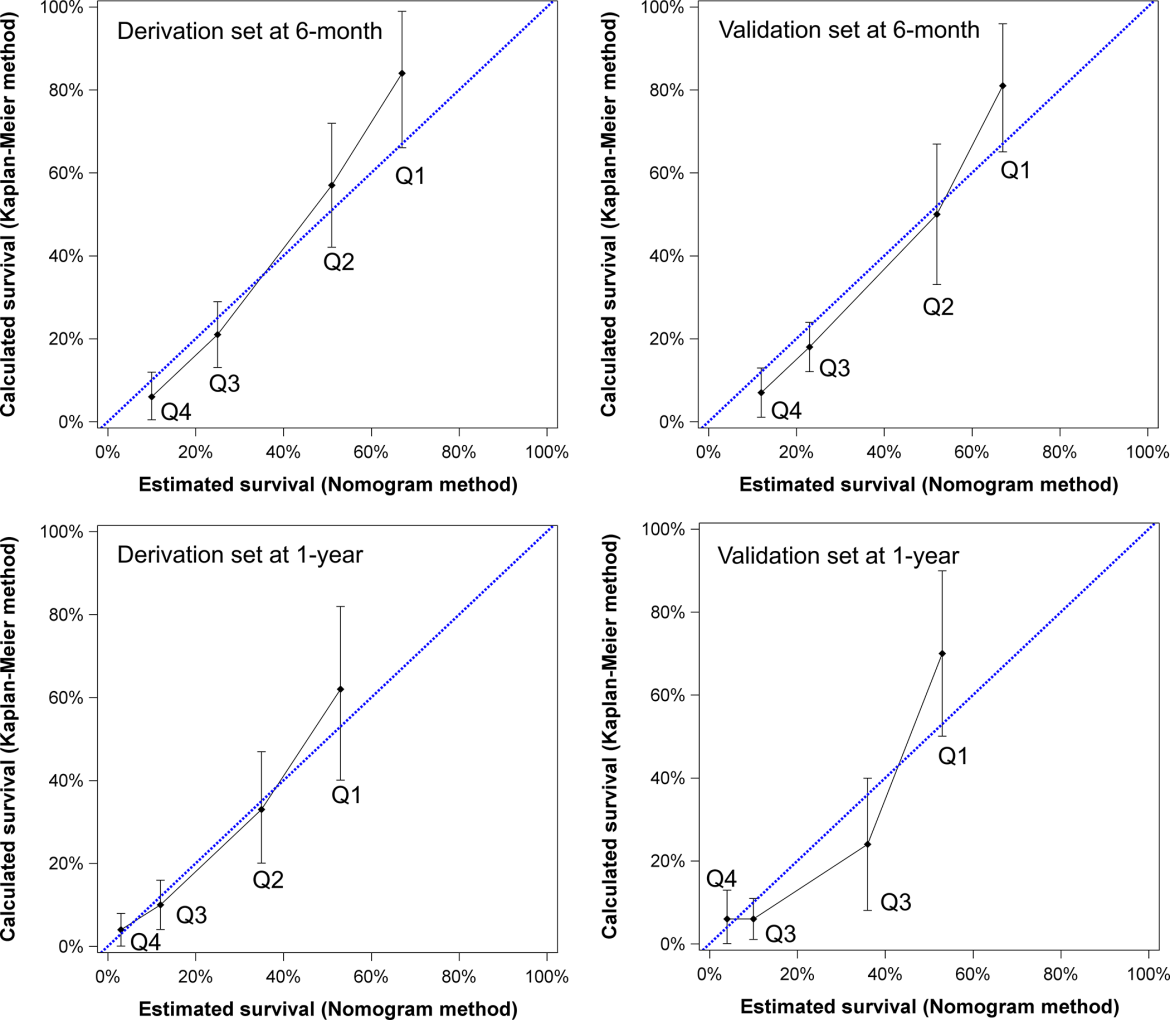
The calibration plots of the nomogram in the derivation and validation sets for 6- and 12-month survival prediction. The X-axis represents the nomogram-predicted survival and the Y-axis shows the mean survival and 95% confidence interval observed by the Kaplan-Meier method. By dividing patients into quarters based on nomogram points, the calibration line fits along with the 45-degree reference for both 6- and 12-month survival prediction in derivation and validation sets.

The nomogram generated from the derivation group had a concordance index of 0.759 (95% confidence interval [CI]: 0.552–0.923). Patients were divided into quarters by their specified points to investigate the accuracy of the model (nomogram points 5.8–12, 12.1–18, 18.1–24, 24.1–30.7). In the calibration plots (Fig 4), the mean and 95% CI of survival rates calculated by using the Kaplan-Meier method are shown on the Y-axis and the mean survival estimated by using the nomogram method is shown on the X-axis. The calibration plots for both 6-month and 1-year survival well matched the 45-degree line for derivation set patients.

### Discrimination and calibration of nomogram in the validation set

For the validation set, the nomogram had a concordance index of 0.741 (95% CI: 0.529–0.913). As shown in Fig 4, the nomogram-predicted mean survival is covered within the 95% CI of Kaplan-Meier method observed mean survival at 6 and 12 months for all quarters.

## Discussion

HCC patients are classified as BCLC stage D due to CTP class C or PS 3–4, which are adapted to serve as sufficient criteria regardless of tumor burden. The arbitrary design of BCLC stage D results in remarkably complex compositions, and currently there are very few data focusing on the prediction of survival in BCLC stage D patients. In this study, we specifically investigated the prognostic effect of tumor burden, and proposed a new nomogram for patients with BCLC stage D HCC. By using clinically available parameters, our findings provide accurate survival estimation for terminal stage HCC based on the individual level, and may potentially improve the currently used staging systems for HCC.[1, 19]

The BCLC staging system is primarily determined by tumor burden, severity of cirrhosis and PS. Tumor burden (Okuda staging) had been a part of BCLC stage D when first published in 1999;[20, 21] however the HCC guidelines recommended by both European Association for the Study of the Liver and America Association for the Study of Liver Diseases removed tumor burden from the criteria of BCLC stage D.[1, 19] Patients classified into BCLC stage D could have very diverse clinical profiles; for example, patients with CTP class C, extra-hepatic metastases, and multiple co-morbidities are considered the same BCLC stage as patients with PS 3, minimal cirrhosis and a small resectable HCC nodule. With a well followed-up HCC cohort in our series, the baseline information of the study patients clearly showed that 15% (59/386) of patients had tumor burden within the Milan criteria. In addition, there were 17% and 9% of patients classified as CTP class A and PS 0–1, respectively. These findings disclose that a substantially high proportion of BCLC stage D patients had relatively small tumor burden, mild cirrhosis or relatively stable general condition at the time of diagnosis, indicating individualized prognostic prediction should be considered necessary from the clinical perspective.

BCLC stage D patients with mild cirrhosis and small tumor burden might potentially benefit from surgical resection or TACE. Similarly, selected patients with CTP class C and small tumor burden could choose liver transplantation or ablation to effectively prolong their survival.[22–24] Tumor burden has been shown an important survival predictor and is also highly related to treatment modalities.[25, 26] In this study, by dividing patients into three categories (within the Milan criteria, with distant involvement and vascular invasion, and the rest), both univariate and multivariate survival analyses showed the excellent discriminating power of tumor burden. Importantly, tumor burden 3 had the highest BETA value in the Cox regression model, which highlights the importance of tumor burden in predicting the clinical outcome. Consistently, the predominant prognostic power of tumor burden was also illustrated in our previous nomogram study for unselected HCC patients.[7] Although tumor burden is not considered a criterion for BCLC stage D HCC, our findings explicitly display the decisive role of tumor burden when the prognostic stratification is specifically evaluated within BCLC stage D.

In addition to tumor burden, cirrhosis and PS, the three parameters of original BCLC system, we found that hepatitis B and high serum AFP level were also associated with a poor prognosis as identified in the prognostic model. HBV infection was reported to associate with high tumor burden; notably, some studies showed HBV-related HCC patients had worse outcome compared to HCC patients without chronic viral hepatitis or patients with HCV-related HCC.[27–29] A multicenter study also pointed out HBV-related HCC patients suffered decreased survival compared to HCV-related HCC patients with matched clinical features.[30] The other factor, serum AFP at a level of > 400 ng/mL, was reported to have significantly discriminating ability for overall survival in HCC patients.[31] Abundant studies have associated aggressiveness of HCC and worse survival in patients with high serum AFP levels.[8, 32, 33] Altogether, these results suggest that our nomogram is a feasible and clinically accessible model in terms of outcome prediction.

The nomogram has concordance indices of 0.759 and 0.741 for derivation and validation sets, respectively. The interpretation of this finding is that if two HCC patients with different nomogram points are selected, the probability that the patient with higher nomogram score would die earlier is around 75%. Calibration plots showed nomogram-predicted survival covered by the 95% CI of mean survival observed by using the Kaplan-Meier method at 6 and 12 months for both derivation and validation sets. Clearly, this nomogram model shows patients with lower nomogram points (less severe cancer stage) had better survival distribution. With this nomogram, BCLC stage D patients could have individualized survival prediction, and candidates for future clinical trials can be more specifically identified.[34, 35]

This study has some limitations. First, the nomogram was generated from a cohort where hepatitis B is the main cause of chronic liver disease. External validation is required before it can be widely used in countries with high prevalence of alcoholic liver disease or hepatitis C. Second, anti-cancer treatments were not included in this study. Further study is needed to clarify the prognostic effect of variable treatment strategies for BCLC stage D patients. Also, only 2% of patients received transplantation in this cohort. For medical centers with a high volume of liver transplantation, this nomogram might not be suitable for survival prediction. Last, hepatitis B and C viral loads and specific anti-viral treatment may affect patient survival; this study does not include these factors because only a minority of patients received anti-viral treatment at different time periods and this nomogram was designed for all patients with different etiologies of HCC. Nomograms focusing on HCC patients with hepatitis B or hepatitis C are required to further investigate the influence of these variables.

In conclusion, contrary to the current BCLC scheme, this study indicates that tumor burden is a pivotal prognostic factor for patients with BCLC stage D HCC. With this easy-to-use nomogram, BCLC stage D patients can be better evaluated and stratified. An improved healthcare strategy can be planned according to the nomogram, which can also serve to identify candidates for anti-cancer treatments in future clinical trials.

## Supporting information

S1 File S1_file.sas7bdat. The minimal anonymized data set.
(SAS7BDAT)

## Abbreviations

AFP: α-fetoprotein
BCLC: Barcelona Clinic Liver Cancer
CTP: Child-Turcotte-Pugh
HCC: hepatocellular carcinoma
eGFR: estimated glomerular filtration rate
HBV: hepatitis B virus
HCV: hepatitis C virus
PS: performance status
TACE: transarterial chemoembolization
